# Individual and collective empowerment and associated factors among Brazilian adults: a cross-sectional study

**DOI:** 10.1186/s12889-015-2113-7

**Published:** 2015-08-12

**Authors:** Marcia Fatima Soares, Rachel Conceição Ferreira, Camila Alessandra Pazzini, Denise Vieira Travassos, Saul Martins Paiva, Efigênia Ferreira e Ferreira

**Affiliations:** Student in Public Health at Faculty of Dentistry, Universidade Federal de Minas Gerais Belo Horizonte, Av. Presidente Antonio Carlos, 6627, Belo Horizonte, 31270-901 Minas Gerais Brazil; University Center Lavras, Rua Benjamin Constant, 33, Bairro Centro, ZIP Code 37200-000 Lavras, Minas Gerais Brazil; Department of Social and Preventive Dentistry, Faculty of Dentistry, Universidade Federal de Minas Gerais, Av. Presidente Antonio Carlos, 6627, Belo Horizonte, 31270-901 Minas Gerais Brazil; Rua João Antonio Cardoso, 46/601, Bairro Ouro Preto, Belo Horizonte, 31330-390 Brazil; Professor of the Graduate Program in General Dentistry, Universidade Vale do Rio Verde, Três Corações, Brazil; Rua Benjamin Constant 33, Bairro Centro, ZIP Code 37200-000 Lavras, Minas Gerais Brazil; Rua Felipe dos Santos 16/700 Bairro de Lourdes, Belo Horizonte, ZIP Code 30180160 Minas Gerais Brazil; Department of Pediatric Dentistry and Orthodontics, Faculty of Dentistry, Universidade Federal de Minas Gerais, Avenida Bandeirantes, 2275/500 Bairro Bandeirantes, 30.210-420 Belo Horizonte, Minas Gerais Brazil

**Keywords:** Empowerment, Social capital, Lifestyle, Health conditions, Quality of life, Adults

## Abstract

**Background:**

The empowerment embedded in the health area is defined as a process that can facilitate control over the determinants of health of individuals and population as a way to improve health. The aim of this study was to verify the association between individual and collective empowerment with sociodemographic conditions, lifestyle, health conditions and quality of life.

**Method:**

A cross-sectional analytical study was conducted with 1150 individuals (aged 35 to 44 years). The empowerment was determined by questions from the Integrated Questionnaire for the Measurement of Social Capital (IQ-MSC). The quality of life was measured using the WHOQOL (World Health Organization Quality of Life-Bref). Lifestyle and health conditions were obtained by adapted questions from the Fantastic Lifestyle Questionnaire The DMFT Index was incorporated in the health conditions questions. Logistic regression or multinomial regression was performed.

**Results:**

The practice of physical activity was related to individual (OR: 2.70) and collective (OR: 1.57) empowerment. Regarding individual empowerment, people with higher education level (5–11 years – OR: 3.46 and ≥12 years – OR: 4.41), who felt more able to deal with stress (OR:3.76), who presented a high score on quality of life (psychological domain) (OR:1.23) and that smoked (OR:1.49) were more likely to feel able to make decisions and participate in community activities. The increase in the DMFT Index represented less chance of individuals to feel more able to make decisions (OR: 0.96). Regarding the collective empowerment, being religious (catholic) (OR: 1.82), do not drink or drink just a little (OR: 1.66 and 2.28, respectively), and increased score of overall quality of life (OR: 1.08) were more likely to report that people cooperate to solve a problem in their community.

**Conclusion:**

The two approaches to empowerment, the individual and collective are connected, and the physical activity showed to be a good strategy for the empowerment construction.

## Background

The Lalonde [1] report published in 1974 is considered a starting point in the worldwide movement of Health Promotion. It brought a new understanding of the determinants of health and the need for more health care actions. In addition to clinical care, there is a need for interventions in the environment, risk moderation and better understanding of the complexity of the individual and their social context.

After this report, the resulting actions regarding investments in lifestyle and self-care brought changes in the 80s. The better understanding of the social context in which human being live, and their influence on behavior change for health, exposes a certain weakness in expected results [2].

Health promotion is seen as the main strategy for reducing morbidity and early mortality. This strengthens one guideline: the individual and collective empowerment to participate actively in the health-building process. With the need to involve all segments of society, the concept of empowerment is incorporated as a centerpiece of Health Promotion [3].

Historically, the term empowerment has its origin in movements for social justice in the 60s, such as the mobilization of blacks, women and homosexuals, all in defense of human rights and social justice [4]. The health empowerment is defined as a process that can facilitate control over the determinants of health as a fundamental strategy for achieving health [3].

Empowerment can occur at two levels: the psychological/individual and the community/collective. The individual level refers to the greater ability of individuals to feel strong to make choices in their lives. The collective empowerment refers to the capacity of a community, through the participation process, to achieve collectively defined goals [5].

An effective empowerment process requires the involvement of individual and collective levels. The community with established social values can influence the lifestyles of its individuals, even among the empowered [6]. The lifestyle is one of the four determinants of health groups [1]. If worked isolated, it does not include social and cultural determinants.

While the empowerment processes is been significantly associated with health outcomes, including self-care behaviors [7, 8], especially if the modifications include a supportive social environment [9, 10], it must not be considered a solution itself and may present positive or negative results [7, 10].

Some studies were dedicated to the empowerment theme, most of them by means to measure social capital, since it is considered an empowerment domain [11]. Dimensions evaluated in these instruments could determinate the capacity to act in own benefit or for the community. This ability was observed [12] on those who were classified with high social capital.

Some authors observed an strong inverse association between collective social capital and dental pain [13], dental caries [14] or dental loss [15], all relating the results to the power of the community.

However, others observed an inverse association between high social capital and benefits to health, such as the small dental care usage of adults with high social capital [16].

Few studies evaluated factors associated with empowerment, which facilitates or make in more difficult its construction. In a study with young refugees who participated in an integration program to newcomer youth in Canada [17], difficulties in the empowerment construction were observed. The authors identified that the sense of belonging, positive self-identity, emotional well-being and the auto-determination could allow or restrict the building of the individual empowerment. These factors can be found in population at different ages, but particularly among the marginalized.

Therefore, this subject is relatively unexplored. To better understand what contributes to the individual and community empowerment construction, the aim of this study was to verify the association between empowerment and sociodemographic characteristics, lifestyle health conditions and quality of life.

## Methods

This study is part of a Project (The oral health of adults in the Metropolitan Region of Belo Horizonte (MRBH): objective and subjective aspects) started in 2010 and developed in the Public Health Graduate Program, at the Faculty of Dentistry of the Universidade Federal de Minas Gerais.

It is a cross-sectional study among adults, male and female, 35 to 44 years old, which is a standard age group for oral health conditions surveillance in adults [18], living in MRBH (32 municipalities).

Belo Horizonte is the capital of the Minas Gerais state, located in the southeast of Brazil and is the 6th largest city and the third largest metropolitan area in Brazil [19]. The MRBH is a political, financial, educational and cultural center of Minas Gerais representing around 25 % of the population and 40 % of the economy of Minas Gerais state.

The method used for sample size calculation was to estimate proportions with a correction for the finite population. The methodology and data collection instruments were tested in a pilot study involving 98 participants with the same age group, randomly selected in one municipality in the region, not included in the study.

The pilot study allowed to verify the distribution of adults in the parameters to be investigated and the issues related to empowerment [20]. The frequency of individuals who responded positively to the items was used in the calculation of the sample size. It was assumed an 80 % power, 5 % significance level and a design effect equal to 2.0, to compensate the minor variation in the cluster sample. The sample was calculated from 758 individuals, with a 20 % addition compensating possible losses, with a total of 934 individuals. Although this is a cross-sectional study, losses could be the fact that individuals could not accept to take part of the study or could not be located at home after three attempts.

For sample selection, it was considered the total of inhabitants in each municipality, grouped according quartiles of population size. Two municipalities in each quartile were randomly selected. The cluster sampling was used to select blocks from municipalities with up to 50 thousand (Cluster I) and census tracts and blocks from municipalities with over 50 thousand inhabitants (Cluster II). The number of adults examined was proportional to the population of the municipality. All residences located in randomly selected blocks were visited.

Data collection was performed by a structured questionnaire in the form of interviews and oral health evaluations in the participant’s households between May and December of 2010. The oral health evaluations were conducted under natural light using mirrors, dental plans and wooden spatulas. The condition of the tooth crown was recorded according to the WHO criteria [18], excluding third molars. This was the only data that composed the “health conditions” variable group.

For the oral health evaluations, an expert performed the theoretical calibration of five researchers, presenting photographs of clinical conditions to be study. The agreement on the clinical examination was tested with 12 volunteers at a dental clinic in a teaching institution, yielding kappa values ranging from 0.81 to 0.92 interobserver, and 0.80 and 1.00 intraobserver.

The dependent variables for analyzes were individual empowerment and collective empowerment, built on issues of the Integrated Questionnaire for the Measurement of Social Capital (SC -IQ) [20]. *The individual empowerment* variable was created from the combination of two questions, components of empowerment dimension and political action: *“in the last 12 months, have you or someone in your household participate in any community activity where people gather to do some work for the benefit of the community?”* (Yes: No), and *“you feel you have the power to make decisions that could change the course of your life?”* (totally unable; able or unable; fully capable).

From the combination of the answers to these two questions, four categories were established in this variable: 1. did not participate in community activities and unable to make a decision; 2. did participate in community activities and feel unable to make a decision; 3. did not participate in community activities and feel able to make a decision; 4. did participate in community activities and feel able to make a decision. Thirty-seven (3.6 %) individuals who were in category two were excluded from data analyzes. Maintaining this category in bivariate and multivariate analyzes, resulted in very imprecise estimates. Another design would need to include this group with sufficient sample of those individuals who participates in community activities and feel unable to make a decision.

*The collective empowerment* was assessed through a question of scale collective action and cooperation *“If there was a problem of water supply in this community, what is the probability that people cooperate to solve the problem?”* This question was selected because it is a common problem in smaller municipalities and an uncomfortable issue to the population. The answer options were grouped into two categories for the variable composition: 1-Unlikely/neither likely nor unlikely; 2-likely.

The intention was to be able to measure the empowerment of the community where the individuals live.

The independent variables considered were: Sociodemographic characteristics: sex (male or female), age (35 to 39 and 40 to 44 years old), total years of education (<4, 5 to 11 years), ethnicity (black/yellow/indigenous/brown or white), marital status (with or without companion), religion (no religious, Catholic or otherwise - Protestant/evangelical/spiritualist/Jehovah witness), per capita income, time residing at this location; Lifestyle: physical activity (very little or no); smoker (very little or no); drinking (very little or no); Health conditions: general health perception (very bad/poor, fair, very good/good); health problem that causes pain (yes or no), ability to handle the stress of everyday life (very little or no), ability to relax and enjoy leisure (very, little or no), presence of toothache in the last three months (yes or no), the average DMFT Index (number of decayed, missing and filled permanent teeth).

Besides these data, we measured the quality of life using the WHOQOL (World Health Organization Quality of Life-Bref) version with 26 items, validated in Brazil [21]: two general questions and 24 questions assessing physical, psychological, social and environmental fields. The quality of life scores were computed with range 4–24 points, and higher scores indicate better quality of life. Fifty-two participants who answered less than 21 questions of the WHOQOL were excluded from the analyzes, following the guidelines for implementation and analyzes of the instrument. Variables of lifestyle related to stress and ability to relax were obtained from questions adapted from the Fantastic Lifestyle Questionnaire [22].

The variable per capita income, time living in the same place, and DMFT Index scores of quality of life were included in the statistical and quantitative analyzes.

Statistical analyzes were conducted using Stata Statistical Software (StataCorp LP version). Initially, was performed a descriptive analyzes to obtain mean, standard deviation and proportions. Univariate analyzes were performed to identify factors associated with individual empowerment and collective factors. Logistic regression and multinomial regression was the performed analyzes. For the final model, only variables associated to empowerment with p < 0.25 were included. All analyzes were performed with design effect correction. The svyset command in Stata was used to analyze data of complex samples, considering the levels of sampling and sample weight svyset sector [pweith pesoamostral =]| |block. All further analyzes were conducted using the svy command.

The Ethics Committee of the Universidade Federal de Minas Gerais, approved this study with the Protocol number CAEE0096.0.203.000–09 on 26/03/2009, and all participants signed an informed consent.

## Results

The sample included 1150 adults, most females (66.63 %), 52.30 % had between 35 and 39 years old; 50.58 % presented five to 11 years of education; 72.72 % were Brown/Black/Yellow/Indigenous; 71.49 % were living with a partner and 48.99 % were Catholic. The average per capita income of the participants were $255 (SE = 49.76) and lived an average of 14.8(SE = 0.70) years in the same location.

Regarding the life style, physical activities were still restricted (61.95 %; no), the alcoholic beverages consumption (61.13 %; no) and smoking (79.1 %; no) were low. In the questions related to health conditions, the participants considered themselves with very bad/poor, fair health (68.49 %), with presence of health problems that caused pain (40.46 %) and toothache in the last six months (24.10 %). Almost fifity seven percent (56.65 %) of the participants self-declared capable to relax and 62.97 % the ability to handle the daily stress The average DMFT Index was 16.91 (SE = 0.27) (Table 1).

**Table 1:** Characterization of the sample according to the independent variables investigated

	Total^a^ % (95 % IC)
Sociodemographic	
Sex (*n* = 1150)	
Male	33.37 (18.90–47.84)
Female	66.63 (52.16–81.10)
Age (*n* = 1150)	
35 e 39	52.30 (48.06–56.54)
40 e 44	47.70 (43.46–51.94)
Years of study (*n* = 1144)	
≤4	41.15 (33.27–49.02)
5 a 11	50.58 (43.99–57.17)
≥12	8.27 (5.10–11.45)
Skin color (*n* = 1067)	
Brown, Black, Asian, and Indigenous	72.75 (67.95–77.55)
White	27.25 (22.45–32.05)
Marital status (*n* = 1143)	
No companion	28.51 (24.71–32.31)
With companion	71.49 (67.69–75.29)
Religion (*n* = 1128)	
No religion	9.20 (5.25–13.15)
Catholic	48.99 (38.31–59.68)
Other Religions (Protestant/evangelicals/spiritualist/Jehovah’s witness)	41.81 (34.31–49.30)
Lifestyle	
Physical activity (*n* = 1095)	
No	61.95 (53.97–69.93)
Little	20.02 (15.25–24.80)
Much	18.08 (13.99–22.07)
Smoke cigarettes (*n* = 1096)	
Much	10.91 (7.9913.82)
Little	9.38 (8.06–10.70)
No	79.71 (76.56–82.85)
Consumption of alcoholic beverages (*n* = 1093)	
Much	5.7 (4.19–7.28)
Little	33.13 (26.38–39.89)
No	61.13 (55.09–67.17)
Health Conditions	
General Health Perception (*n* = 1148)	
Very bad/bad	68.49 (60.15–76.83)
Regular	26.80 (20.44–33.15)
Very good/good	4.75 (2.23–7.19)
Have a health problem that causes pain (*n* = 1090)	
Yes	40.46 (35.19–45.73)
No	59.54 (54.27–64.81)
Ability to handle the stress of every day life (*n* = 1121)	
No	8.52 (6.39–10.66)
Little	28.51 (25.11–31.91)
Much	62.97 (58.66–67.28)
Relax and enjoy leisure time (*n* = 1129)	
No	13.46 (11.54–15.38)
Little	29.89 (26.03–33.75)
Much	56.65 (52.27–61.03)
Toothache in the last three months (*n* = 1116)	
Yes	24.10 (20.95–27.63)
No	75.90 (72.74–79.05)

The median and interquartile range of quality of life scores were: physical domain (16.57, 3.43), psychological (15.32, 2.80), social (16.0, 4.0), environment (13.0, 3.0), total score of quality of life (16.0, 2.0).

Regarding individual and collective empowerment, most adults reported that even if they felt able to make a decision to change their course of life and declared non-participation in community activities (59.82 %), 62.91 % reported that the community probably would collaborate on the water supply problem (Table 2).

**Table 2:** Distribution of adults regarding the categories of individual and collective empowerment

	Number	Percent	95 % IC
Individual empowerment			
Did not participate in community activities, feels unable to make decisions that change the course of life	241	22.15	17.80–26.50
Did not participate in community activities, feel able to make a decision that changes the course of life	633	59.82	56.44–63.21
Participated in community activities, feels able to make decisions that change the course of life	178	18.03	13.37–22.68.
Total	1052^a^		
Collective empowerment			
Unlikely collective cooperation + nor likely, nor unlikely	421	37.09	31.14–43.04
Likely collective cooperation	662	62.91	56.96–68.86
Total	1083^b^		

^a^Loss of 98 individuals: 52 excluded because they answered less than 21 questions of the WHOQOL + 37 who answered participate in community activities and feel unable to make decisions that change the course of life + 9 adults did not answer the questions giving rise to individual empowerment variable. ^b^Loss of 67 individuals: 52 excluded because they answered less than 21 questions of the WHOQOL + 15 adults did not answer the question giving rise to collective empowerment variable

The results of descriptive analyzes among independent variables and individual and collective empowerment, and the *deff* associated with each estimate is shown in Table 3. Regarding the choice for deff = 2, the sample representativeness is confirmed once it was sufficient for all variables, with a minor loss only to the relax and enjoy leisure time variable (deff = 2.21).

**Table 3:** Distribution of adults regarding the individual and collective empowerment and independent variables

Independent variables	Individual Empowerment (%)				Collective Empowerment (%)		
	Did not participate/Feels unable	Did not participate/Feels able	Participate/ Feels able	*Deff*	Unlikely + neither likely nor unlikely	Likely	Deff
Sociodemographic							
Sex							
Male	17.31	64.87	17.83		37.40	62.60	
Female	24.58	57.30	18.12	1.20	36.47	63.53	1.25
Age							
35 and 39	21.47	60.30	18.23		36.23	63.77	
40 and 44	22.90	59.29	17.80	1.55	38.04	61.96	0.54
Years of study							
≤4	29.86	58.15	11.99		36.63	63.37	
5 a 11	17.29	60.78	21.93		37.48	62.52	
≥12	13.60	62.46	23,93	1.77	36.65	63.35	1.06
Skin color							
Brown, Black, Asian, and Indigenous	21.75	61.45	16.80		38.85	61.15	
White	23.56	57.71	18.73	1.15	34.47	65.53	0.65
Marital status							
No companion	22.42	57.34	20.24		35.63	64.37	
With companion	22.07	60.64	17.29	1.95	40.92	59.08	1.41
Religion							
No religion	32.09	55.08	12.83		46.79	53.21	
Catholic	21.47	61.96	16.57		31.05	68.95	
Other Religions (Protestant/evangelicals/spiritualist/ Jehovah’s witness)	20.70	58.38	20.92	2.01	41.33	58.67	1.27
*Lifestyle*							
Physical activity							
No	26.29	57.67	16.05		39.35	60.65	
Little	20.08	61.49	18.43		38.31	61.69	
Much	10.28	65.52	24.20	1.56	29.19	70.81	1.08
Smoke cigarettes							
Much	17.13	59.39	23.48		40.02	59.98	
Little	22.12	54.77	23.11		41.14	58.86	
No	22.77	60.42	16.81	1.33	36.34	63.66	1.73
Consumption of alcoholic beverages							
Much	32.51	46.68	20.81		52.55	47.45	
Little	17.87	66.88	15.25		39.06	60.94	
No	23.74	56.80	19.46	1.94	34.81	65.19	1.14
*Health Conditions*							
General Health Perception							
Very bad/bad	18.52	62.36	19.12		34.93	65.07	
Regular	30.67	52.72	16.61		42.54	57.46	
Very good/good	26.57	63.31	10.11	1.21	37.08	62.92	1.21
Have a health problem that causes pain							
Yes	28.32	54.43	17.25		37.40	62.60	
No	18.10	63.69	18.21	1.94	36.99	63.01	0.95
Ability to handle the stress of every day life							
No	42.95	47.23	9.82		44.34	55.66	
Little	30.35	54.25	15.40		35.58	64.42	
Much	15.56	63.79	20.65	2.25	37.04	62.96	1.92
Relax and enjoy leisure time							
No	38.94	45.50	15.56		37.33	62.67	
Little	25.63	57.84	16.53		34.80	65.20	
Much	16.45	64.28	19.27	1.52	38.45	61.55	2.21
Toothache in the last three months							
Yes	25.18	57.68	17.14		36.41	63.59	
No	21.19	60.50	18.31	1.25	37.34	62.66	1.25
Per capita income (average,, EP)	404,79 (50,17)	421,89 (55,46)	432,74 (50,68)	-	410,71 (58,75)	428,73 (48,2)	-
Time to live in the same location (Average, EP)	14,91 (0,68)	13,84 (0,63)	15,19 (1,64)	-	13,13 (0,90)	15,01 (0,83)	-
DMFT (Average, EP)	18,36 (0,38)	16,54 (0,32)	16,77 (0,74)	-	17,08 (0,66)	16,83 (0,31)	-

In the bivariate analyzes, variables associated with individual empowerment with p < 0.25 were: sociodemographic (years of schooling, time living in the same place, religion), lifestyle (physical activity, smoking, alcohol consumption), health (perceived health, ability to cope with stress, to relax and enjoy leisure), quality of life (physical, social, environmental, psychological and overall) and DMFT Index.

The multiple analyzes (Table 4), demonstrated that individual empowerment was positively associated in the two categories used (did not participate/feel capable) to higher education, plenty physical activity, tabagism, more ability to handle the daily stress and quality of life (psychic domain) and DMFT Index (only non set analyzes). The more DMFT the less the chance to feel capable to make decisions to change the course of life.

**Table 4:** Crude and Adjusted model of factors associated with individual empowerment (*n* = 1027)

	Did not participate, feel capable				Participated feel capable			
	OR gross (95 % IC)	p-value	OR adjusted (95 % IC)	p-value	OR gross (95 % IC)	p-value	OR adjusted (95 % IC)	p-value
Socio demographic								
Years of study								
<4	1		1		1		1	
5 a 11	1.80 (1.35–2.41)	<0.001	1.91 (1.22–3.00)	0.007	3.16 (1.31–7.62)	0.012	3.46 (1.16–10.28)	0.027
>12	2.36 (1.40–3.98)	0.002	2.35 (1.03–5.36)	0.043	4.38 (2.10–9.16)	<0.001	4.41 (1.47–13.21)	0.010
*Life style*								
Physical activity								
No	1				1			
Little	1.40 (0.85–2.29)	0.177	1.08 (0.71–1.64)	0.703	1.50 (0.79–2.87)	0.207	1.20 (0.47–3.04)	0.696
Much	2.90 (1.57–5.36)	0.001	2.10 (1.24–3.56)	0.008	3.86 (1.90–7.79)	0.001	2.70 (1.37–5.31)	0.006
Smoke cigarettes								
Much	1		1		1		1	
Little	0.71 (0.31–1.65)	0.417	0.49 (0.17–1.42)	0.179	0.76 (0.31–1.90)	0.547	0.47 (0.20–1.14)	0.091
No	0.77 (0.50–1.18)	0.001	0.47 (0.28–0.78)	0.006	0.54 (0.32–0.91)	0.023	0.26 (0.16–0.43)	<0.001
*Health conditions*								
*Ability to handle the stress of every day life*								
No	1		1		1		1	
Little	1.63 (0.90–2.93)	0.101	1.37 (0.69–2.69)	0.352	2.22 (1.60–8.13)	0.218	1.86 (0.48–7.27)	0.356
Much	3.73 (1.94–7.17)	<0.001	2.49 (1.24–4.95)	0.012	5.81 (2.93–11.51)	<0.001	3.76 (1.67–8.48)	0.002
*Quality of life*								
Psychological domain	1.25 (1.10–1.43)	<0.001	1.20 (1.08–1.33)	0.002	1.23 (1.06–1.43)	0.008	1.23 (1.10–1.43)	0.008
*DMFT*	0.96 (0.93–0.99)	0.042	0.96 (0.92–0.99)	0.042	0.96 (0.91–1.02)	0.187	0.96 (0.91–1.02)	0.221

^a^Results of the multinomial logistic regression. Reference category: not participated/incapable. Final model adjusted for sex and per capita income. The results of crude analyzes of the variables in the final model were presented

The same factors, except the DMFT Index, were associated with greater participation in community activities and the increased feeling of being able to make decisions that change the course of life. However, OR values are higher indicating the additional association of the independent variables on participation in community activities (Table 4).

The variables associated with collective empowerment (p < 0.25) in the univariate analyzes were skin color, marital status, religion, physical activity, alcoholic consumption, general health perception, overall quality of life, social and environmental domains of the quality of life.

The multiple model remained significantly associated with collective empowerment: to be Catholic, do plenty physical activity, do not ingest alcoholic beverage or just a little, and higher overall quality of life (Table 5).

**Table 5:** Crude and Adjusted model of factors associated with collective empowerment. (*n* = 1048)

	OR Gross	Value of p	OR adjusted^a^	Value of p
*Socio demographic variables*				
Religion				
No religion	1		1	
Catholic	1.95 (1.06–3.6.1)	0.034	1.82 (1.15–2.90)	0.013
Protestant/evangelicals/spiritualist/ Jehovah’s witness (other religions)	1.25 (0.76–2.04)	0.361	1.04 (0.72–1.51)	0.820
*Lifestyle*				
Physical activity				
No	1		1	
Little	1.05 (0.74–1.48)	0.796	1.06 (0.69–1.61)	0.795
Much	1.57 (1.09–2.28)	0.019	1.57 (1.12–2.20)	0.012
Consumption of alcoholic beverages				
Much	1		1	
Little	1.73 (1.11–2.69)	0.017	1.66 (1.08–2.54)	0.022
No	2.07 (1.24–3.46)	0.007	2.28 (1.24–4.19)	0.010
*Quality of life*				
Social domain	1.06 (102–1.10)	0.003		
Environmental domain	1.07 (1.00–1.16)	0.059		
Quality of life (Total)	1.09 (1.04–1.16)	0.002	1.08 (1.02–1.52)	0.014

^a^Results of binary logistic regression analyzes. Reference category is Improbable + Neither likely nor unlikely that people cooperate to solve the problem of water supply in the community. The results of crude analyzes of the variables in the final model were presented

## Discussion

Measuring empowerment of individuals and communities is challenging. The option to use a questionnaire as a tool was considered feasible and possible and was the choice in this study. The metropolitan region of Belo Horizonte was excluded from this study sample due of the size (approximately 2.5 million inhabitants) and the peculiarities of a large urban center, showing the highest social indicators compared to other municipalities in the region [19].

Since the sample selection was made according to population size (two municipalities in each quartile), in the 32 municipalities of this study were included municipalities with less than nine thousand inhabitants (quartile 1) and municipalities with more than seventy thousand (quartile 4).

The demographic characteristics of our study sample, is representative of the Brazilian population standard [11, 23], not only in the range size of the population, but regarding aspects such as a high percentage of women in the study sample (66.63 %), lower education among adults (91.73 %; <12 years old), minority of White people (27.25 %), living with a partner (71.49 %) and average per capita income of less than $250 (Table 1).

The study population could be considered with residence stability since it presents an average of 14.8 (SE = 0.70) years living in the same location [19]. This is a considerable time that probably allowed the population to bond socially, which has to be considered when analyzing empowerment, especially collective empowerment.

The participants consider themselves with poor/very poor health (68.49 %), and that could be explained by the higher presence of health problems that caused pain (40.46 %) and dental pain (24.10 %) in the last six months. Signs and symptoms are significant in the self-health evaluation.

The results related to the participation in community activities and in the variable to be able to decide about its own life, demonstrates that 77.83 % of the participantes feelt empowered to solve their personal issues but only for 18.03 %, the empowerment helped them to be involved with the community. However, when it was discussed a practical issue (lack of water supply), which is common to several Brazilian municipalities, the participants revealed the possibility of a considerable community participation (62.91 %).

### Individual and collective Empowerment and its association

Table 6 briefly shows the observed significant associations.

**Table 6:** Individual and Collective Empowerment and associated factors: a conceptual framework

Independent variables associated	Individual Empowerment	Collective Empowerment
Sociodemographic	Higher education	Catholic
Lifestyle	Much Physical Activity	
	Smoker	Do not consume or consume little alcoholic beverage
Health Conditions	Much ability to handle stress	
Quality of life	Domain psychological	All areas

Among the sociodemographic variables used in this study, after multivariate analyzes, education was associated with individual empowerment and religiosity with collective empowerment. Individuals with more years of education compared to those with less education had a higher chance (aged 5–11 years and >12 years) to feel able to make decisions. The education influence cognitive resources, understanding of information and knowledge [24].

The Catholic religion was associated with collective empowerment. In the literature, studies have associated religion to individual empowerment. For the behaviour of prevention and cure of diseases, religion can influence values, lifestyle, cognitions, emotions, and behaviours. The power of faith has demonstrated an important facilitator of individual empowerment, however, for the collective empowerment; limitations and potential negative influences of religion in community settings are discussed. Beliefs and values may negatively affect the decision-making power, but religion has a good capacity to mobilize human and institutional positively or negatively resources. The association between empowerment and religion can be more related with group involvement than with the choice of the religious belief [25–27].

Regarding the lifestyle, physical activity was associated to individual and collective empowerment. Although there is a policy guideline of the Brazilian Ministry of Health from 2011, creating a Health Fitness Program (Programa Academia da Saúde) [28], the practice of physical activity among adults is not an installed habit. In this study, 61.95 % reported no physical activity at work or as a sport; this is a common practice for only 18 % of this population. The men reported more active with 54.2 % practicing physical activity. Among women, the percentage is 29.9 % (p < 0.001).

The municipalities encourage physical activity installing the Health Fitness Program in organized public spaces. All the municipalities included in the study when data collection occurred had one or more centers for physical activity (http://www.mg.gov.br). This is not a routine practice for the predominant gender in this sample (female) that could be explained by culture, poverty, social construction of gender or biological determinism [29].

The smoking habit was associated with individual empowerment. No smokers (79.42 %) were less likely to participate in community activities and to felt able to make important decisions for their life or their community. The research PETab: Brazil Report, published in 2011 by the National Cancer Institute - INCA in partnership with the Pan American Health Organization -PAHO reported a significant decline of smokers in Brazil (34.8 % in 1989 and 18.2 % in 2008) and a lower percentage of smokers among women, prevalent gender in our sample. Furthermore, there is a population awareness about smoking in Brazil, with a decrease of social acceptability of smokers [30]. Tobacco control has provoked among smokers a concern about the degree of harm that may be caused to passive smokers [31–33]. The anti-smokers norms influence social relationships. According to the Brazil report [30], the decrease of smoking is less in population groups with lower income and schooling, similar to this study population. The association observed in this study may be related to the psycho-emotional sensations caused by this habit. According to participants of smoking cessation activities, smoking calms, divert, gives pleasure, relieves the sadness and decreases anxiety. It is understandable the smoking habit association with the individual empowerment and no association with the collective empowerment [34].

The less or no consumption of alcoholic beverage (94,26 %) was associated with collective empowerment. Alcoholic beverage use was strongly associated with disarrangement and violent behavior than sociability or social support [35, 36].

Regarding the health conditions variable, only the item ability to handle stress (62.97 %) was associated to individual empowerment, both for those who participated in community activities and for those who did not. Studies have demonstrated an association between stress caused by daily work [37, 38] and need to know how to deal with it, diminishing its negative effects on health and quality of life.

A measure of the quality of life was also common for individual and collective empowerment. For the individual, there was a significant association only with the psychological dimension related to aspects such as self-esteem, body/image appearance, feelings, and others. In the collective level, a correlation to the total index Lifestyles (WHOQOL) with all domains (physical, psychological social and environmental) were observed. A study of institutionalized elderly with nursing care demonstrated an association between quality of life and empowerment perceived by this group [39].

The major limitation of this study is the cross-sectional methodology measuring subjective questions with quantitative methods. Since it is an underexplored theme, identifying hypothesis could bring significant value to new evaluations and empowerment guidance.

### Conclusions

This study pointed out several hypotheses about the empowerment of the community. The data presented, and the observed associations lead us to some reflections directly related to training for the individual and collective empowerment.

The existence of an association between empowerment and physical activity, whether at an individual or collective level make us belive that such practice of health promotion must be seen as a valuable ally in the construction of empowerment.

Behaviorist approach that demonize sedentary, blaming the individual, showing the physical activity to reduce the epidemiological condition, solely [6] can ward off the individual from this habit with prejudice to the construction of health empowerment. Approaches that consider the physical activity can bring benefits to individuals and communities

Individual empowerment was also associated with the higher educational level, to the capacity to handle stress and to the psychological domain of Quality of Life Index. These are plausible associations since it clarifies issues such as self-esteem and well-being were the first conditions for individual action.

Collective empowerment was associated with religion, no consumption of alcoholic beverages and to have a good quality of life. The lack of association of collective empowerment with the higher level of education is also the point of concern regarding the empowerment of individuals and community. The education, in Peter Demo words, “is the incubator of citizenship”. This is a known association and one of the critical empowerment points.

The issue of smoking and its association with individual empowerment needs a better reflection. Despite government initiatives to control these behaviors, there is a great investment by the smoking companies in advertising, that in a way offers the power, pleasure, and happiness. This publicity is uneven, with high investment in more vulnerable people [4], disseminating a false power. The fact that smoking was associated with empowerment in this study, confirm the false power felt by a drug consumption that is a nonhealthy habit.

The two approaches to empowerment, the individual and collective were connected and the physical activity showed to be a good strategy for the building of empowerment

It reinforces the need to understand individuals in their social context and the weakness to expect changes in behavior and lifestyle, with the sole effort aimed at changing behavior. This aspect is fundamental in a training of individuals and communities.
